# Effect of colonoscopy screening on the risk of colorectal cancer in China: a follow-up study

**DOI:** 10.3389/fonc.2025.1689066

**Published:** 2025-11-12

**Authors:** Yunxin Kong, De Liu, Yue Ma, Zongmei Dong, Yiran Wang, Xiaohu Luo, Hongying Zhao, Rui Jin, Siyuan Gao, Guihua Zhang, Dong Dong, Pan Zhang, Lang Zhuo

**Affiliations:** 1 Cancer Prevention Office, Xuzhou Cancer Hospital, Xuzhou, China; 2 Department of Control and Prevention of Chronic Noncommunicable Diseases, Xuzhou Center for Disease Control and Prevention, Xuzhou, China; 3 Gastroenterology Department, Xuzhou Cancer Hospital, Xuzhou, China; 4 Clinical Laboratory, Xuzhou Cancer Hospital, Xuzhou, China; 5 Department of Medical Oncology, Xuzhou Cancer Hospital, Xuzhou, China; 6 School of Management, Xuzhou Medical University, Xuzhou, China; 7 School of Public Health, Xuzhou Medical University, Xuzhou, China

**Keywords:** colorectal neoplasm, mass screening, colonoscopy, incidence, mortality

## Abstract

**Background:**

Colorectal cancer (CRC) is one of the most common cancers worldwide. Colonoscopy is the gold standard for CRC screening, but its effectiveness in population-based programs requires further evaluation.

**Methods:**

We conducted a follow-up study in Xuzhou, China. Participants were recruited from 2014 to 2021, with follow-up continuing until December 2023. The study comprised two components: 1) an active follow-up to assess treatment outcomes for patients with colorectal advanced neoplasia (CAN) detected during screening; 2) a passive follow-up to compare CRC incidence and mortality between participants who underwent colonoscopy and those who refused it.

**Results:**

The active follow-up included 196 participants, while 15,440 were included the passive follow-up (4,029 in the colonoscopy group and 11,411 in the non-colonoscopy group). 96.43% (189/196) CAN patients were actively followed. However, only 25.93% (49/189) received treatment. The CRC incidence density was 35.77 per 100,000 person-years in the colonoscopy group, which was significantly lower than the 95.50 per 100,000 in the non-colonoscopy group (*IRR* = 0.37, *P* = 0.011). 83.33% (5/6) of the CRC cases in the colonoscopy group were from the subgroup of CAN patients who did not receive treatment. There was no significant difference in CRC mortality between the two groups.

**Conclusions:**

Colonoscopy screening is effective in reducing the risk of CRC. However, its real-world effectiveness has been compromised by the low participation rate and the poor treatment adherence among screen-positive patients. The impact of colonoscopy screening on reducing CRC mortality remains undetermined.

## Background

Colorectal cancer (CRC) is one of the most common cancers in the world. More than 1.9 million new cases of CRC and 904,000 deaths are estimated to have occurred in 2022, representing close to one in 10 cancer cases and deaths, ranking third in terms of incidence but second in terms of mortality (1). In China, the incidence and mortality of CRC have increased rapidly in recent years, with 517,100 new cases of CRC and 240,000 deaths in 2022; CRC ranks second in terms of cancer incidence and fourth in terms of cancer mortality and has become an urgent public health issue (2).

Most CRC cases are considered to occur through the “adenoma-carcinoma” pathway, which usually lasts 5-10 years (3, 4). Therefore, screening and early intervention have been clearly demonstrated to be effective in preventing occurrence and improving survival rates of CRC (5–7). Various screening tests are available, each with their own advantages and disadvantages and varying levels of evidence to support their use, but high-quality evidence to indicate the best strategies is limited (7–11). The most commonly used screening methods are fecal occult blood tests and endoscopic screening with sigmoidoscopy or colonoscopy.

In current research and clinical practice, colonoscopy is the widely adopted gold standard for CRC screening. During the procedure, the endoscopist can thoroughly examine the entire colon and rectum through the visual lens, and for any suspicious lesions found, tissue biopsy can be performed to further clarify the pathological diagnosis. Other than colonoscopy, all screening tests are “2-step” tests: if the result is abnormal, a follow-up colonoscopy is required. The search and evaluation team for the China Guideline for the Screening, Early Detection and Early Treatment of Colorectal Cancer (2020,Beijing) (11) conducted a systematic review in 2020 to evaluate the effectiveness of colonoscopy screening in reducing the incidence and mortality rates of CRC in the population, and the analysis results showed that compared with no screening, colonoscopy screening could reduce the risk of disease by 56% and the risk of death by 57%. However, in a 10-year follow-up randomized controlled trial conducted in Poland, Norway, Switzerland and New Zealand (NordICC), colonoscopy screening only reduced the incidence of CRC by 18%, and had almost no effect on reducing the CRC mortality rate (12). The effectiveness of colonoscopy screening in the general population may still require further research for validation (13).

In order to determine the effectiveness of colonoscopy screening in China, we conducted a follow-up study within the framework of the Cancer Screening Program in Urban China (CanSPUC) in Xuzhou. From July 2014 to December 2021, colonoscopy screenings were conducted in Xuzhou. Xuzhou is the central city of the Huaihai Economic Zone (which has a population of 119 million, covers an area of 178,000 km^2^ and consists of 20 cities), located at the junction area of four provinces (Jiangsu, Anhui, Shandong and Henan), southeast of the North China Plain, gateway to East China. The follow-up consists of two parts. The follow-up team of the hospital conducts active follow-up to assess the treatment outcomes of colorectal advanced neoplasm (CAN) after screening. The follow-up team of the disease control center obtains the incidence and mortality of CRC among those who have undergone colonoscopy and those who have refused colonoscopy through passive follow-up, and makes a comparison.

## Methods

### Study design and population

We conducted a follow-up study under the framework of CanSPUC (14). CanSPUC is an ongoing national cancer screening program in urban areas of China and Xuzhou joined the program in August 2014. Briefly, a cluster sampling method was adopted to conduct simple random sampling with the community as a group in the main urban area of Xuzhou. Residents living in selected communities aged 40-74 years were approached by trained staff via phone calls and personal encounters. After obtaining signed written informed consent, all the eligible participants (age 40-74 years old, local permanent resident population, no major diseases) were interviewed by trained staffs to collect information about their exposure to risk factors and to evaluate their cancer risk using conditions set by the National Cancer Center. To optimize the use of limited colonoscopy resources and to increase the detection rate of colorectal neoplasia, only participants who met the high-risk conditions for CRC were recommended to undergo colonoscopy at Xuzhou Cancer Hospital, which is designated by the programmer free of charge. All data collection processes were conducted via an information system built specifically for CanSPUC by the National Cancer Center.

**Figure 1:**
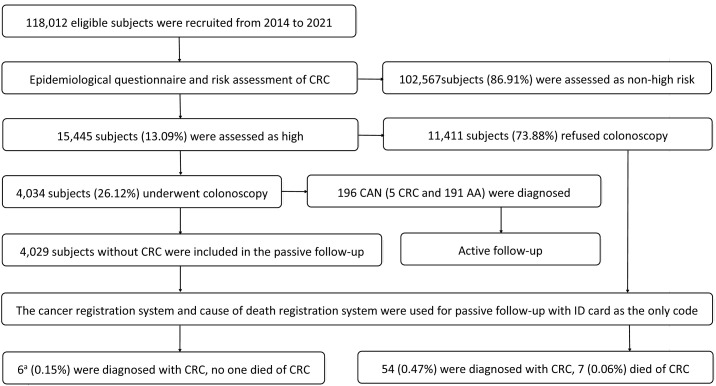
Recruitment and follow-up flow chart. CRC: colorectal cancer; CAN: colorectal advanced neoplasm; AA: advanced adenoma. ^a^5 case detected by colonoscopy screening at baseline was not included.

From July 2014 to December 2021, CRC screening was conducted in Xuzhou, a total of 118,012 participants were recruited and completed epidemiological questionnaires. Among them, 15,445 subjects (13.09%) at high risk for CRC were invited to undergo colonoscopy, and 4,034 (26.12%) subjects underwent colonoscopy. This screening identified 196 cases (4.86%) of CAN, who were enrolled in an active follow-up to monitor treatment outcomes. The 15,440 high-risk participants without CRC were included in a passive follow-up, utilizing the city’s cancer and cause of death registration systems (with ID card as the unique identifier). This passive cohort was divided into two groups based on screening adherence: the colonoscopy group (n=4,029) and the non-colonoscopy group (n=11,411). The recruitment and follow-up flow chart is shown in **Figure 1**.

### Ethics approval and consent

This study was approved by the Ethics Committee of Xuzhou Cancer Hospital (approval number: 2023-02-030-K01). All participants provided written informed consent prior to enrollment. The consent process comprehensively covered both the initial screening procedures (including colonoscopy) and the subsequent use of their data for long-term follow-up and analysis.

### Colonoscopy screening and active follow-up

Participants at high risk for CRC were recommended to undergo colonoscopy at Xuzhou Cancer Hospital, which was designated by the programmer free of charge. The average time from completing the questionnaire to undergoing colonoscopy was 17.05 days. The nature, benefits and risks of colonoscopy were explained to all the subjects prior to the examination, and a colonoscopy risk notification form was signed. We used polyethylene glycol (HYGECONR, Jiangxi Hygecon Pharmaceutical Co., Ltd., China) as a standard bowel preparation regimen for all participants, and an electrocardiogram was also performed before colonoscopy to prevent unexpected events. A team of experienced physicians, colorectal surgeons, nurses and anaesthetists performed all the colonoscopy procedures at the endoscopy center of Xuzhou Cancer Hospital. All abnormal findings were pathologically examined in accordance with clinical procedures, and the results and images were uploaded to the project information system. CAN was the most important abnormal finding and was defined as CRC or any colorectal adenoma measuring 1 cm or more in diameter, high-grade dysplasia, or tubular-villous histologic features. To ensure the quality of the examination, the quality control team, composed of the chief physician and the deputy chief physician, reviewed all the results.

Among the 4,034 participants who participated in the colonoscopy screening, 196 individuals (4.86%) were diagnosed with CAN (including 5 CRC). Gastroenterologists informed the patients of their screening results and provided treatment recommendations, while the subsequent treatment decisions were made by the patients themselves. For all patients diagnosed with CAN during screening, the hospital follow-up team conducted active follow-ups at 3 months, 6 months, and annually after the screening. These follow-ups assessed subsequent treatment status, documented reasons for treatment refusal, and provided recommendations for re-examination and treatment.

### Sample size for passive follow-up

We calculated the sample size under the assumption that the CRC incidence density in the colonoscopy group was lower than that in the non-colonoscopy group. The average enrollment age of the study subjects in the non-colonoscopy group was 58 year old, and the incidence of CRC in the Chinese population aged 55-60 years in 2020 was approximately 100 per 100,000 (15), suppose the risk of CRC in the high-risk population is 150% of that in the average-risk population, so the estimated CRC incidence density in the non-colonoscopy group was 150 per 100,000. Assuming that colonoscopy screening can reduce the incidence of CRC by 56% (11), the CRC incidence density is estimated to be 66 per 100,000 in the colonoscopy group. The per capita person-years of observation were expected to be 5 years, and the ratio of the number of people in each group was 1:3 in the colonoscopy group and the non-colonoscopy group. On the basis of these assumptions, at the *P* < 0.05 significance level, to achieve 80% power to detect the difference in CRC incidence density between groups, 3,321 and 9,963 patients were required in the colonoscopy group and non-colonoscopy group. The actual number of participants in each group was 4,029 and 11,411, which met the sample size requirements.

### Passive follow-up and end point

All participants were passively followed from their completion of risk assessment (the colonoscopy group began by completing the colonoscopy screening) at CanSPUC until December 2023. The individual follow-up time was the difference between the end time of follow-up and the start time of follow-up. The follow-up time for each participant was summed to derive the total person-years. The cancer registration system and cause of death registration system of Xuzhou were used for passive follow-up, with ID card as the only code.

The primary endpoints were the risk of CRC and death from CRC. The secondary endpoint was death from any cause. A diagnosis of CRC was defined, according to the International Statistical Classification of Diseases and Related Health Problems, 10th revision, as cancer in the colon or rectum (topography codes C18 to C20, combined with International Classification of Diseases for Oncology morphology codes for adenocarcinoma). The stage of CRC was classified as stage I, stage II, stage III, stage IV, or unknown according to the Pathological TNM stage (the 8th edition of AJCC cancer staging system). CRC-related deaths were defined as those that were listed as such in the cause of death registries.

### Statistical analysis

Statistical analysis was performed with Stata 17.0. A two-tailed P value of <0.05 was considered statistically significant. Baseline characteristics of the study population were summarized and compared between the colonoscopy and non-colonoscopy groups. Qualitative data are presented as numbers (percentages) and were compared using the Pearson *χ^2^* test or Fisher’s exact test, as appropriate.

Person-years of follow-up were calculated for each participant from the entry date (colonoscopy group from the date of colonoscopy completion, non-colonoscopy group from the date of baseline risk assessment completion) until the date of an endpoint event (CRC diagnosis, CRC death, or all-cause death) or the censoring date (December 31, 2023), whichever occurred first. The follow-up time for each individual was calculated in years (days/365.25), and the total person-years for each group were derived by summing these individual follow-up times.

The incidence and mortality densities for the colonoscopy and non-colonoscopy groups were calculated separately. The CRC incidence density, CRC mortality density, and all-cause mortality density were each calculated according to the formula: Density = (Number of events/Total person-years) × 100,000, where “events” corresponds to new CRC cases, deaths from CRC, and deaths from any cause, respectively.

The incidence rate ratio (*IRR*) of CRC incidence density, CRC death density and all-cause death density for the colonoscopy group compared to the non-colonoscopy group was calculated directly. Due to the low number of events, multivariate adjustment was not performed, as it could lead to unstable estimates.

To address the potential for immortal time bias arising from the differential start of follow-up (colonoscopy group from the date of colonoscopy completion, non-colonoscopy group from the date of baseline risk assessment completion), we conducted a sensitivity analysis. In this analysis, the start of follow-up for the colonoscopy group was re-defined as the date of baseline risk assessment completion, aligning it with the non-colonoscopy group. Incidence densities and the *IRR* were then recalculated based on this unified definition.

## Results

### Characteristics of participants

The study population, recruited from July 2014 to December 2021, was divided into two cohorts based on baseline screening results. The active follow-up cohort included 196 participants who screened positive for CAN, and their baseline characteristics are presented in **Supplementary Table S1**. The passive follow-up cohort included 15,440 participants who were free of CRC at baseline. This cohort was further subdivided into a colonoscopy group (4,029; 26.10%) and a non-colonoscopy group (11,411; 73.90%). The comparative baseline characteristics of these two groups are detailed in **Table 1**, which shows statistically significant differences in all measures (P < 0.001).

**Table 1:** Characteristics of the participants in passive follow-up (%).

Characteristics	Colonoscopy group (n=4,029)	Non-colonoscopy group (n=11,411)	*P*
Sex			
Male	1,918 (47.60)	6,946 (60.87)	<0.001
Female	2,111 (52.40)	4,465 (39.13)	
Enrollment age (years)			<0.001
40-44	124 (3.08)	551 (4.83)	
45-49	608 (15.09)	1,191 (10.44)	
50-54	835 (20.72)	1,612 (14.13)	
55-59	902 (22.39)	1,935 (16.96)	
60-64	715 (17.75)	1,669 (14.63)	
65-69	648 (16.08)	2,619 (22.95)	
70-74	197 (4.89)	1,834 (16.07)	
Family history of CRC (first degree relatives)			<0.001
No	3,656 (90.74)	10,679 (93.59)	
Yes	373 (9.26)	732 (6.41)	
Cigarette smoking (Current or past)			<0.001
No	2,277 (56.52)	6,829 (59.85)	
Yes	1,752 (43.48)	4,582 (40.15)	
BMI (kg/m^2^)			<0.001
<23	892 (22.14)	2,019 (17.69)	
≥23	3137 (77.86)	9,392 (82.31)	
Enrollment time			<0.001
2014	267 (6.63)	1,217 (10.67)	
2015	289 (7.17)	1,512 (13.25)	
2016	241 (5.98)	1,104 (9.67)	
2017	420 (10.42)	1,154 (10.11)	
2018	110 (2.71)	401 (3.51)	
2019	324 (8.04)	455 (3.99)	
2020	487 (12.09)	1,151 (10.09)	
2021	1,896 (46.96)	4,417 (38.71)	

### Active follow-up

96.43% (189/196) of the patients with CAN in the colonoscopy group received active follow-up. The results showed that among the patients who received active follow-up, only 25.93% (49/189) underwent treatment (including all 5 CRC patients) (**Supplementary Table S2**). The subgroup analysis showed that the treatment rates were higher in female (31.67%) and those aged 45-49 (45.45%), 50-54 (40.00%) and 70-74 (68.00%), while the rates were lower in male (23.26%) and those aged 55-59 (13.51%), 60-64 (11.90%), 65-69 (14.58%).

The main reason for refusing treatment was that the patients themselves believed that no treatment was necessary, and this accounted for 86.43% (121/140) of the cases. Other reasons for refusing treatment include lack of time (12.86%) and inability to afford the treatment costs (0.71%).

### Incidence density of colorectal cancer

By the end of passive follow-up in December 2023, the average follow-up time was 4.75 years (73316.30/15440) and the median follow-up time was 3.16 years (the shortest is 0.16 years, while the longest is 9.50 years). A total of 60 subjects were diagnosed with CRC, and the incidence densities of CRC were 35.77 per 100,000 (6/16,773.29) and 95.50 per 100,000 (54/56,543.01) in the colonoscopy group and the non-colonoscopy group. The incidence density of CRC in the colonoscopy group was lower than that in the non-colonoscopy group (*IRR* = 0.37, 95%*CI*: 0.13-0.87, *P* = 0.011). Subgroup analysis showed that colonoscopy screening significantly reduced the incidence density of CRC in male (*IRR* = 0.38, 95%*CI*: 0.10-1.06, *P* = 0.045) and subjects aged 50-59 (*IRR* = 0, 95%*CI*: 0-1.14, *P* = 0.037) (**Table 2**).

**Table 2:** The CRC incidence density in each group.

Group	CRC	Follow-up person-year	CRC incidence density (per 100,000, 95%*CI*)	*IRR* (95%*CI*)	*P*
Colonoscopy group	6	16,773.29	35.77 (13.14-77.88)	0.37 (0.13-0.87)	0.011
Non-colonoscopy group	54	56,543.01	95.50 (71.86-124.82)	1	

Sex					
Male					
Colonoscopy group	4	7896.63	50.65 (13.81-129.68)	0.38 (0.10-1.06)	0.045
Non-colonoscopy group	40	30181.26	132.53 (94.61-181.41)	1	
Female					
Colonoscopy group	2	8876.66	22.53 (2.73-81.38)	0.42 (0.05-1.85)	0.254
Non-colonoscopy group	14	26361.76	53.11 (29.04-89.12)	1	

Enrollment age (years)					
40-49					
Colonoscopy group	1	3278.62	30.50 (0.77-169.99)	0.59 (0.01-4.84)	0.694
Non-colonoscopy group	6	11550.71	51.95 (19.06-113.10)	1	
50-59					
Colonoscopy group	0	6945.34	0 (0-53.11)^a^	0 (0-1.14)	0.037
Non-colonoscopy group	11	19893.51	55.29 (27.62-98.91)	1	
≥60					
Colonoscopy group	5	6549.33	76.34 (24.79-178.13)	0.52 (0.16-1.32)	0.154
Non-colonoscopy group	37	25098.80	147.42 (103.85-203.27)	1	

a one-sided, 97.5% confidence interval.

CRC: colorectal cancer.

A sensitivity analysis, which aligned the start of follow-up for both groups at the date of risk assessment to address potential immortal time bias, yielded a nearly identical *IRR* of 0.37 (95%*CI*: 0.13-0.86, *P* = 0.011) (**Supplementary Table S3**).

### Colorectal cancer incidence at different follow-up times

In the colonoscopy group, 5 CRC cases were diagnosed within 1-2 year after the screening and 1 case of CRC was diagnosed within 5-6 year after the screening, 83.33% (5/6) were confirmed as CAN during colonoscopy screening and did not receive treatment. In the non-colonoscopy group, the incidence of CRC was higher in the first three years and the last two years of follow-up. The incidence of CRC in the colonoscopy group was lower than that in the non-colonoscopy group during the follow-up periods of 0-1 year and 2-3 years. There was no statistically significant difference in the incidence of CRC between the two groups during the remaining time (**Supplementary Table S4**).

### Stages of colorectal cancer

Among the 60 patients diagnosed with CRC, 39 (65.00%) had a pathological diagnosis, and 21 (35.00%) did not choose to be seen locally, did not receive treatment or did not have a pathological diagnosis (**Supplementary Table S5**). In cases with pathological staging, 75% (3/4) of the patients in the colonoscopy group and 60% (21/35) of those in the non-colonoscopy group were in the early stage (stage I and stage II), there was no statistically significant difference between the two groups (*P* = 0.548).

### Mortality density

The CRC mortality density in the colonoscopy group and the non-colonoscopy group were 0 (one-sided, 97.5%*CI*: 0-21.73 per 100,000) and 12.35 per 100,000 (95%*CI*: 4.97 per 100,000-25.44 per 100,000) respectively, and the difference was not statistically significant (*IRR* = 0, 95%CI: 0-2.32, *P* = 0.160) (**Table 3**). The all-cause mortality density were 294.60 per 100,000 (95%*CI*: 218.64 per 100,000-390.24 per 100,000) and 651.16 per 100,000 (95%*CI*: 586.00 per 100,000-721.31 per 100,000) respectively. The mortality density in the colonoscopy group was lower than that in the non-colonoscopy group (*IRR* = 0.45, 95%*CI*: 0.33-0.61, *P* < 0.001).

**Table 3:** The all-cause and CRC death density in each group.

CRC mortality density					
Group	CRC death	Follow-up person-year	CRC mortality density (per 100,000, 95%*CI*)	*IRR* (95%*CI*)	*P*
Colonoscopy group	0	16972.37	0 (0-21.73)^a^	0 (0-2.32)	0.160
Non-colonoscopy group	7	56668.97	12.35 (4.97-25.44)	1	
All-cause mortality density					
Group	All-cause death	Follow-up person-year	All-cause mortality density (per 100,000, 95%*CI*)	*IRR* (95%*CI*)	*P*
Colonoscopy group	50	16972.37	294.60 (218.64-390.24)	0.45 (0.33-0.61)	<0.001
Non-colonoscopy group	369	56668.97	651.16 (586.00-721.31)	1	

a one-sided, 97.5% confidence interval.

CRC: colorectal cancer.

## Discussion

In this study, we determined the effectiveness of colonoscopy screening in Xuzhou, China. The study found that colonoscopy screening is effective in reducing the risk of CRC. However, the impact on reducing CRC mortality has not been determined.

The most important finding of this study is that colonoscopy screening is effective in reducing the incidence of CRC, and the CRC incidence density was reduced by 63% in the colonoscopy group compared with the non-colonoscopy group, consistent with the results of the China Guideline for the Screening, Early Detection and Early Treatment of Colorectal Cancer (2020,Beijing) (reduce the risk of CRC by 56%) (11), better than the results of NordICC (subgroup analysis of those who had the colonoscopy done showed that the risk of developing from CRC decreased by approximately 30%) (12, 13). Further analysis indicates that colonoscopy screening primarily reduced the risk of CRC in males and individuals aged 50-59. This might mainly be caused by two reasons. On the one hand, the incidence of CRC among females and individuals aged 40-49 was relatively low (11, 15), and longer follow-up may be required to determine the screening effectiveness in these groups. On the other hand, for participants over 60 years old, the low CAN treatment rate may explain the absence of CRC risk reduction. A 20-year cohort study from the United States shows that participants with CAN were significantly more likely to develop CRC compared with those with no adenomas (*SHR* = 3.24, 95%*CI*: 2.32-4.52) (16). In this study, among the 6 CRC cases identified during follow-up in the colonoscopy group, 5 originated from untreated CANs, with 4 of these patients being over 60 years old.

The primary goal of CRC screening is the early detection of lesions, enabling timely intervention. The effectiveness of screening in reducing CRC risk depends critically on both the identification and subsequent treatment of CAN. In the past, we believed that the desire of almost all patients to have their polyps removed immediately upon discovery was very strong (17). Therefore, most research has focused on CAN detection rates (18–20), the treatment rate of identified adenomas has received considerably less attention. However, merely having a high detection rate is not sufficient. If it cannot be effectively linked with effective treatment measures, then the preventive effect of the screening will be greatly diminished. In this study, although both the gastroenterologists and the hospital’s follow-up team were involved in the active follow-up of CAN patients, the treatment rate was only approximately 26%. This low treatment rate led to an increase in CRC incidence shortly after screening and compromised both early diagnosis and treatment rates. The primary reason for refusal was patients’ belief that treatment was unnecessary—a perception likely attributable to the typically asymptomatic nature of CAN. Since whether to receive treatment is decided by the patients themselves, more effective health education measures may be needed to change their attitudes.

The participation rate is also a key factor influencing the effectiveness of colonoscopy screening, and it is one of the major challenges faced by countries around the world. For instance, in the NordICC trial conducted in Europe, the participation rate of colonoscopy ranged from 22.9% (the Netherlands) to 60.7% (Norway) (21). Even in the United States, where CRC screening was introduced earlier, colonoscopy participation was only 60.8% (22). The colonoscopy participation rate of the high-risk population in this study was 26.12%, which was higher than the national participation rate of 14% (10) but still far from the ideal participation rate. Low colonoscopy compliance can be attributed to a number of barriers, such as pain, embarrassment, and a lack of awareness of screening (10, 21, 23). Sedated colonoscopy can address problems of pain and embarrassment, thereby improving colonoscopy compliance, but it significantly increasing screening costs (24). Providing alternative screening methods, such as FIT, to participants who decline colonoscopy, and then recalling those with positive results for colonoscopy screening, may also be an effective way to increase colonoscopy participation rates. In the TARGET-C study conducted in China, the colonoscopy participation rate among FIT-positive subjects was higher than that of those directly assigned to colonoscopy screening (25, 26).

The impact of colonoscopy screening on reducing colorectal cancer (CRC) mortality was not established in this study. This is likely attributable to the relatively short median follow-up period of 3 years, which may be insufficient to observe significant mortality benefits. In previous studies, in order to observe the impact of screening methods on the mortality rate of CRC, it is usually necessary to conduct follow-up observations for a period of 10 years or longer (12, 16). To address this limitation and evaluate the long-term efficacy of colonoscopy screening in reducing CRC mortality, continued follow-up of our study population is planned.

An interesting finding of our study was the statistically significant reduction in all-cause mortality without a concomitant significant difference in CRC mortality. This apparent discrepancy may be explained by several non-mutually exclusive factors. First, the act of undergoing colonoscopy itself may serve as a proxy marker for overall better health awareness and access to healthcare (22, 27). Individuals who are compliant with screening recommendations may generally exhibit healthier behaviors (e.g., balanced diet, regular exercise, non-smoking), have higher socioeconomic status, and be more engaged with the healthcare system for other preventive services. These factors are associated with lower risks of major causes of death beyond CRC, such as cardiovascular disease and other cancers (28, 29). Consequently, the screened group might have had a baseline advantage in overall health, contributing to the observed all-cause mortality benefit. Second, the role of competing risks must be considered. In an aging population, the risk of dying from other common causes (e.g., heart disease) may be substantially higher than the risk of dying from CRC. If the screening intervention, through the mechanisms described above, reduced mortality from these more prevalent conditions, it could generate a detectable signal in all-cause mortality even if its absolute effect on CRC-specific death was modest.

This study has several strengths. First, to our knowledge, this is the first population study in Xuzhou, China to verify the effectiveness of colonoscopy screening on the risk of CRC. Second, in addition to the passive endpoint assessment, we also actively tracked and documented the treatment outcomes of individuals with positive screening results. This provided crucial background information for interpreting the results. Third, this study was conducted under the framework of CanSPUC, which uses rigorous standards to guarantee the integrity and accuracy of the collected data, including a review mechanism to ensure the quality of the data and the development of a data system to monitor all the processes of the study.

This study also has several limitations. First, due to logistical constraints, CanSPUC was implemented only in urban Xuzhou, limiting the generalizability of our findings to rural populations. Second, the 3-year median follow-up was sufficient to assess the early effect of screening on CRC incidence but remains too short to observe a mortality benefit, which requires longer-term study. Third, significant baseline differences between groups indicate potential self-selection bias. The small number of CRC events precluded adjusted analysis; thus, the reported incidence rate ratio reflects overall effectiveness(which may overestimate the pure efficacy of colonoscopy) rather than a bias-adjusted effect. These limitations underscore that the real-world impact of screening depends not only on its technological efficacy, but also on broad, equitable participation and sustained follow-up. At last, the differential start of follow-up between groups could theoretically introduce immortal time bias, although our sensitivity analysis showed its impact on the incidence estimate was minimal.

In summary, colonoscopy screening is effective in reducing the risk of CRC, but challenges remain with low participation rates and poor treatment adherence among screen-positive individuals. Due to the relatively short average follow-up period, the impact of colonoscopy screening on reducing CRC mortality has not been determined.

## Data Availability

The original contributions presented in the study are included in the article/**Supplementary Material**. Further inquiries can be directed to the corresponding authors.
