# Supplementary material for: Effect of colonoscopy screening on the risk of colorectal cancer in China: a follow-up study
Source: Front Oncol. 2025 Nov 12;15:1689066. doi: 10.3389/fonc.2025.1689066 (PMC12646884; doi:10.3389/fonc.2025.1689066)
Supplement: Supplementary file 1 [file Table1.doc]

Table S1. Characteristics of the participants in active follow-up (n=196)

| **Characteristics** | **N (%)** | **Characteristics** | **N (%)** |
| --- | --- | --- | --- |
| **Sex** |  | **Cigarette smoking (Current or past)** |  |
| Male | 136 (69.39) | No | 79 (40.31) |
| Female | 60 (30.61) | Yes | 117 (59.69) |
| **Enrollment age (years)** |  | **BMI (kg/m^2^)** |  |
| 40-44 | 1 (0.51) | <23 | 35 (17.86) |
| 45-49 | 11 (5.61) | ≥23 | 161 (82.14) |
| 50-54 | 27 (13.77) | **Enrollment time** |  |
| 55-59 | 39 (19.90) | 2014 | 7 (3.57) |
| 60-64 | 43 (21.94) | 2015 | 14 (7.14) |
| 65-69 | 50 (25.51) | 2016 | 6 (3.06) |
| 70-74 | 25 (12.76) | 2017 | 13 (6.63) |
| **Family history of CRC (first degree relatives)** |  | 2018 | 5 (2.55) |
| No | 181 (92.35) | 2019 | 11 (5.61) |
| Yes | 15 (7.65) | 2020 | 32 (16.33) |
|  |  | 2021 | 108 (55.10) |

Table S2. Active follow-up and treatment outcomes of participants with colorectal advanced neoplasm.

| **Characteristics** | **Colorectal advanced neoplasm (n=196)*** | **Received active follow-up (n=189)*** | **Active follow-up rate (%)** | **Received treatment (n=49)*** | **Treatment rate (%)** |
| --- | --- | --- | --- | --- | --- |
| **Sex** |  |  |  |  |  |
| Male | 136 | 129 | 94.85 | 30 | 23.26 |
| Female | 60 | 60 | 100 | 19 | 31.67 |
| **Enrollment age (years)** |  |  |  |  |  |
| 40-44 | 1 | 1 | 100 | 0 | 0 |
| 45-49 | 11 | 11 | 100 | 5 | 45.45 |
| 50-54 | 27 | 25 | 92.59 | 10 | 40.00 |
| 55-59 | 39 | 37 | 94.87 | 5 | 13.51 |
| 60-64 | 43 | 42 | 97.67 | 5 | 11.90 |
| 65-69 | 50 | 48 | 96.00 | 7 | 14.58 |
| 70-74 | 25 | 25 | 100 | 17 | 68.00 |

* Including 5 cases of colorectal cancer, 4 were male and 1 was female; 2 were aged 65-69, and 3 were aged 70-74.

Table S3. Sensitivity Analysis of the Incidence Rate Ratio Using a Unified Start of Follow-up

| **Group** | **CRC** | **Follow-up person-year** | **CRC incidence density (per 100,000, 95%*CI*)** | ***IRR* (95%*CI*)** | ***P*** |
| --- | --- | --- | --- | --- | --- |
| Colonoscopy group | 6 | 16961.38 | 35.37 (12.98-76.99) | 0.37 (0.13-0.86) | 0.011 |
| Non-colonoscopy group | 54 | 56,543.01 | 95.50 (71.86-124.82) | 1 |  |

Table S4. The CRC incidence in each group at different follow-up time.

| **Follow-up time (year)** | **Colonoscopy group** | | |  | **Non-colonoscopy group** | | | ***P*** |
| --- | --- | --- | --- | --- | --- | --- | --- | --- |
|  | **CRC** | **No. of subject** | **Incidence**  **(per 100,000, 95%CI)** |  | **CRC** | **No. of subject** | **Incidence**  **(per 100,000, 95%CI)** |  |
| 0-1 | 0 | 4029 | 0 (0-91.52^a^) |  | 12 | 11411 | 105.16 (54.35-183.62) | 0.007 |
| 1-2 | 5 | 4026 | 124.19 (40.34-289.58) |  | 9 | 11366 | 79.18 (36.21-150.26) | 0.431 |
| 2-3 | 0 | 4014 | 0 (0-91.86^a^) |  | 14 | 11284 | 124.07 (67.85-208.08) | 0.004 |
| 3-4 | 0 | 2125 | 0 (0-173.44^a^) |  | 5 | 6915 | 72.31 (23.48-168.66) | 0.102 |
| 4-5 | 0 | 1637 | 0 (0-225.09^a^) |  | 2 | 5759 | 34.73 (4.21-125.39) | 1.000 |
| 5-6 | 1 | 1310 | 76.34 (1.93-424.57) |  | 3 | 5283 | 56.79 (11.71-165.86) | 0.588 |
| 6-7 | 0 | 1196 | 0 (0-307.96^a^) |  | 2 | 4866 | 41.10 (4.98-148.39) | 1.000 |
| 7-8 | 0 | 777 | 0 (0-473.63^a^) |  | 2 | 3728 | 53.65 (6.50-193.66) | 1.000 |
| 8-9 | 0 | 540 | 0 (0-680.80^a^) |  | 3 | 2637 | 113.77 (23.47-332.11) | 1.000 |
| ≥9 | 0 | 252 | 0 (0-1453.18^a^) |  | 2 | 1175 | 170.21 (20.62-613.50) | 1.000 |

^a^ one-sided, 97.5% confidence interval.

CRC:colorectal cancer

Table S5. The Pathological TNM stage of CRC in each group.

| **Stage** | **Colonoscopy group (%)** | **Non-colonoscopy group (%)** |
| --- | --- | --- |
| Ⅰ | 3 (50.00) | 12 (22.22) |
| Ⅱ | 0 | 9 (16.67) |
| Ⅲ | 1 (16.67) | 8 (14.81) |
| Ⅳ | 0 | 6 (11.11) |
| Unknown* | 2 (33.33) | 19 (35.19) |

* Including patients who do not choose to be seen locally, do not receive treatment or do not have a pathological diagnosis.
